# Investigating the support for equitable admissions policies in health professions education: the Formal Consensus method

**DOI:** 10.1186/s12909-024-06049-y

**Published:** 2024-10-17

**Authors:** Lianne Mulder, A. Wouters, S. Somra, A. S. Koster, J. H. Ravesloot, G. Croiset, R. A. Kusurkar

**Affiliations:** 1 Amsterdam UMC location Vrije Universiteit Amsterdam, Research in Education, De Boelelaan 1118, Amsterdam, The Netherlands; 2 LEARN! Research Institute for Learning and Education, Faculty of Psychology and Education, Vrije Universiteit Amsterdam, Amsterdam, The Netherlands; 3 The Hague Municipal Health Services, The Hague, The Netherlands; 4 Department of Pharmaceutical Sciences, Utrecht University, Utrecht, The Netherlands; 5 Amsterdam UMC location University of Amsterdam, Medical Biology, Meibergdreef 9, Amsterdam, The Netherlands; 6 Wenckebach Institute for Education and Training, University Medical Center Groningen, Groningen, The Netherlands

**Keywords:** Educational policy, Equitable admissions, Formal consensus method, Contextualized Admissions, Lottery, Diversity

## Abstract

Higher education institutions increasingly aim to implement equity in admissions. However, there is no one-size-fits-all solution to determine which equitable admissions procedures are suitable in a specific context, nor which groups should be its beneficiaries. Therefore, we applied the Formal Consensus Method (FCM) to investigate the support amongst experts and stakeholders for different equitable admissions policies and target groups within the context of Health Professions Education in The Netherlands. We found majority support (median of ratings ≥ 7 on a scale of 1 to 9) in both groups for the target groups ‘applicants with a low or average socio-economic status’ and ‘applicants with an underrepresented migration background’. The majority of participants was also in favor of Contextualized Admissions, especially when used to increase enrolment of applicants with a low or average socio-economic status, with an underrepresented migration background, and asylum status holders. Lastly, both groups supported lottery with extra tickets for applicants with an underrepresented migration background. However, as the range of ratings fell outside the FCM prescribed range of 5-9, no case in which there was majority support could be defined as a ‘consensus’. The expert group proposed the use of Bonded Medical Places for applicants from the Caribbean parts of the Dutch Kingdom. The policies and target groups for which broad support was found, could contribute to equitable admissions, improved student diversity, and enhanced quality of health education and future care. Our application of the FCM in the area of equitable admissions policies may be useful for researchers in other countries where equity principles are not (widely) used in admissions decisions.

**Supplementary Information:**

The online version contains supplementary material available at 10.1186/s12909-024-06049-y.

## Background of the study

There is increasing attention for promoting diversity, equity and inclusion in selection and admissions in higher education [1, 2]. Since performance on selection instruments can be biased by applicants’ sociodemographic characteristics [3–5], many higher education institutions (HEIs) are working to develop equitable admissions procedures. Their aims can be to improve access to higher education for applicant groups which are underrepresented and/or face structural disadvantages in entering higher education [3, 6, 7], to increase social mobility [8], and to admit a representative student population [9–11]. This is especially important in Health Professions Education (HPE), as a diverse and representative (future) workforce is essential in achieving excellent healthcare for all patient populations [12–14].

Each country has its unique history of inequities in education, leading to accumulated disadvantages for e.g. youth of lower socioeconomic status (SES) backgrounds and/or with an underrepresented ethnic background [15–21]. This results in the underrepresentation of different groups in HEIs [1, 22–24]. Each context is therefore unique, and different geographical and educational contexts may benefit from different types of admission procedures to promote equitable admissions for a variety of target groups [25–27]. Equitable admissions policies are sometimes disputed in the legislative arena (e.g. court cases against Affirmative Action [28] or the prohibition of ‘race-conscious’ admissions policies in parts of the U.S [29, 30]. Political considerations thus have an effect on selection practices [31].

The degree to which stakeholders consider selection tools and procedures as appropriate or acceptable (political validity) is important to consider in designing equitable admissions [32]. Simultaneously, there is no consensus on what makes admissions equitable, what constitutes merit [33], or how ‘fair’ assessment in equitable admissions is fostered [34–37]. For example, some consider lottery – rather than selection – as fair [38], whereas others see the use of contextual information to advance equity in admissions as fair [39]. The lack of consensus shows the need for a consensus method to investigate equitable admissions policies which are suitable in a specific context, evidence-based *and* supported by HEIs and society at large. Therefore, this article describes how we applied the Formal Consensus Method (FCM) to identify suitable equitable admissions policies in HPE. The FCM [40] is developed by the French High Authority of Health, and is normally used to reach consensus in the development of evidence-based protocols and policies in healthcare. It asks experts and stakeholders to rate evidence-based proposals on a scale of 1 to 9, with 1 meaning ‘totally unsuitable’ and 9 meaning ‘totally suitable’. The method has predetermined guidelines on when consensus about proposals is formally reached. There needs to be majority support (median ≥ 7) and the range of ratings should fall between 5-9. To the best of our knowledge, the FCM has not been previously applied to the field of (health professions) education. This method may prove to be useful in higher education admissions contexts, in HPE and beyond, where policy changes need to be supported by not only scientific evidence, but also by experts and stakeholders in society. For this paper, we investigated the societal and institutional support amongst experts and stakeholders for different equitable admissions policies and target groups in HPE in The Netherlands. While different types of selection procedures may vary in the extent to which they contribute to equality of opportunity or equity, our research focuses on policies which are explicitly aimed at increasing the representation of underrepresented students in HPE.

### The global need for equitable admissions in Health Professions Education

In many countries, the HPE student population is not representative of the general population [41–44]. This is problematic, because diversity amongst HPE students and health professionals is of fundamental importance for the provision of excellent education and healthcare to all patient populations including in underserved areas, improved culturally-sensitive care and improved research into diseases which primarily affect patients of color [12, 15, 16, 45–47]. Additionally, there is a need to select for a diversity in knowledge, skills and attributes, because different HPE specialties may require different competencies in professionals [31, 48]. Lastly, students who belong to groups who are disadvantaged in society, due to e.g. discrimination and poverty, bring competencies, knowledge and skills to healthcare about the social hierarchies that contribute to unequitable health conditions [49]. Their lived experiences can improve the connection with their patients and colleagues, and their perspectives can impact healthcare policies, thereby contributing to achieving equitable health outcomes [33, 50, 51]. However, in the selection procedures for HPE programs, not all applicant groups have the same odds of admission [5, 6, 52–56]. The inequality of opportunity in admissions contributes to the matriculation of an HPE student population which does not reflect the diversity in the patient population they will serve in the future.

To address educational inequities and the limited representativeness of the student population, HPE programs across the world have developed different admissions procedures that attempt to promote equity [23, 57–60]. In such procedures, they may use different types of sociodemographic and contextual data of applicants [8], in order to make admissions decisions which increase the matriculation of underrepresented students, and thereby improve the quality of the educational experience at their institution and the future healthcare of all patients [13]. These existing types of equitable admissions, targeted at different groups of underrepresented populations in the health professions, provided the basis for our present study.

### The research setting: university-level Health Professions Education in The Netherlands

Aspiring HPE students in The Netherlands mostly apply to university in their final year of high school. The Dutch high school system is tracked, and university applicants need to have completed the pre-university track (‘vwo’, 6 years). Some study programs use selection procedures, due to capacity limitations. Each selective program can design their own selection procedure. Legally, they must use at least two types of qualitative selection criteria (e.g. intelligence, motivation, study skills) [61], resulting in institutional differences [62].

Students of university HPE programs in The Netherlands are disproportionately female (>70%), and disproportionately have at least one parent with a top-10% income (>60%), compared to their age peers (33%) [54]. When The Netherlands gradually transitioned from a lottery-based to a selection-based admission system, the inequality of opportunity in admission increased significantly. In a 100% selection-based procedure, applicants with at least one parent who was a registered health professional had significantly higher odds to be admitted (odds ratio [OR] and 95% confidence interval: 1.27 [1.10–1.47]), just like female applicants (OR 1.35 [1.19–1.53]) and applicants belonging to the top-10% wealthiest households (OR 1.28 [1.10–1.49]). Applicants with a Turkish, Moroccan, Surinamese or Dutch Caribbean migration background (part of the largest groups of (children of) migrants in The Netherlands) have significantly lower odds to be admitted (OR 0.72 [0.57–0.91]), which could not be explained by the income or wealth levels of their parents. If the existing inequality of opportunity is left unaddressed, the future healthcare professional workforce will become even less representative of the increasingly diverse patient population [54, 63]. More recent research in the Dutch higher education context also has shown that the inequality of opportunity in selection is widespread, and that migration background is the most influential factor in this inequality [56]. Intersectional analyses have shown large differences in the chances of being admitted to a selective higher education program. For example, in 2019, 48.4% of female Medicine applicants without a migration background received an offer of admission (1504/3107), in contrast to male Medicine applicants with a Turkish, Moroccan, Surinamese, Dutch Caribbean or Indonesian migration background: only 32% of this group received such an offer (81/253) [56]. A qualitative interview study with aspiring HPE students in Dutch high schools also indicated that background can influence opportunities to adequately prepare for the selection procedure, especially because having access to a social network connection in the medical field is an important resource which is unequally distributed [64].

Using a lottery-based admissions system (used in the Netherlands until 2017) has been legalized again in 2023 to increase equal opportunities and improve student diversity. However, research shows that a lottery will not suffice in achieving a representative student population in HPE programs, due to a lack of diversity in the applicant pool. Men, applicants from lower and average socio-economic status backgrounds, and applicants with a Turkish, Moroccan, Surinamese, Dutch Caribbean and Indonesian migration background are underrepresented compared to their age peers. Lottery will therefore, at best, result in a student population representative of the applicant pool: approximately 70% female, and disproportionately from high-income backgrounds. Thus, other, more radical measures, seem required. Furthermore, research shows that Dutch applicants to undergraduate HPE programs are not in favor of lottery-based admission [65].

The inequality of opportunity for different groups of (aspiring) applicants, and the limited diversity of the applicant pool, together provide ground for the implementation of equity-promoting admission procedures which take applicants’ sociodemographic backgrounds into account. However, many such policies which are common internationally (such as quota and Contextualized Admissions), are currently not permitted by the Dutch law. This means that demographic background information of applicants, such as sex, migration background, socio-economic indicators, or other factors, cannot be taken into account when making admission decisions or evaluating/contextualizing applicants’ achievements. Furthermore, it is not known whether such policies would be supported by Dutch experts and stakeholders. This potential support is important, as Dutch admissions policies have been influenced for many years by public opinion [66]. Therefore, we aimed to answer the following research question*: What is the level of societal and institutional support in The Netherlands for different equitable admissions policies and target groups in Health Professions Education programs?* The answer to this research question could inform possible legislative changes that make equitable admissions possible.

## Method

We chose the FCM [40] to investigate the current level of support for different potential equitable admissions policies and target groups. The FCM enables the combination of input of experts in a particular field and that of stakeholders in the society at large, on a range of policy matters where consensus is sought. We chose this method after comparison with other consensus methods such as the Delphi method, nominal group technique (NGT) and the RAND/UCLA Appropriateness Method [67]. While the other methods focus on the input of one group of people (usually subject matter experts), the FCM also incorporates stakeholders in a meaningful and impactful way. Thereby, it enabled us to include the views of relevant organizations and groups of people who may not be subject experts, but who could be affected by the proposed policies, if implemented. We thus found it to be the most suitable method to answer the research question, as we were interested in both the institutional and societal support for equitable admissions policies. The FCM uses a convergent design, meaning that quantitative and qualitative data are gathered and analyzed at the same time [68].

### Participants

#### Steering group

In the FCM, the steering group ideally consists of 6-8 professionals who draft the review of the scientific evidence and the proposals. L.M. (sociologist and PhD student) was project manager and chairperson of the steering group. S.S. (MSc Health Sciences) was the research assistant. A.W., A.S.K., J.H.R., G.C. and R.A.K. supervised the research, assisted in the recruitment of experts and stakeholders, and provided feedback on all research documents. A.W. (assistant professor) has expertise in the field of (selection for) medical education and diversity research. A.S.K. (associate professor) was director of education of a Pharmacy programme with a diverse student population. J.H.R. (professor) was director of a medical school in a multi-ethnic community. G.C. (professor) is dean of education and training of an academic medical center, and has experience in admissions and diversity research. R.A.K. (professor, research program leader) has experience in diversity research in medical education. L.M., A.W., S.S., A.S.K., J.H.R., and G.C. were first generation students. S.S. and R.A.K. have a South Asian migration background. L.M., A.W., A.S.K., J.H.R. and G.C. are white Dutch without a migration background. All authors share a mutual interest in the subject of equitable opportunities in HPE. The diversity of our backgrounds encouraged reflexivity [69] and critical dialogue, ensured we evaluated different approaches towards the research project design and execution, and resulted in proactively looking for potential blind spots in our data collection and analysis.

#### Expert group

The expert group is normally expected, in the FCM, to consist of 9-15 subject matter experts. Since the equitable admissions policies which we proposed (see Table 1) have never been legal in The Netherlands, it was not possible to invite experts of equitable admissions in Dutch HPE. Therefore, we chose to invite persons with expertise and experience in selection for HPE programs, diversity in healthcare, and/or diversity in higher education, to have a combined expertise which was relevant in our study. Experts were recruited using purposive sampling [70], and invited via e-mail and telephone. The group consisted of two physicians with expertise in diversity in healthcare, one expert of diversity policy in higher education, and fourteen representatives of all Dutch faculties of Medicine, Clinical Technology, Pharmacy and Dentistry which use selection-based admission. These representatives were selection committee members, program directors and/or HPE selection researchers.

**Table 1:** Equitable admissions policy types

Contextualized Admissions (CA)	In CA (also called ‘whole context review’ or ‘holistic review’ [71]), sociodemographic data about applicants are used to evaluate or weigh their previous achievements (e.g. GPA, test scores) in light of structural factors which may have influenced their performance (e.g. SES, type of high school, first generation at university, etc.) [6, 58, 72, 73], thereby considering the context in which results were achieved. These data are used to assess whether an applicant has the potential to be successful in higher education, even if e.g. their test scores are not as high as that of other applicants [8, 74]. Some universities compare an applicant’s GPA to the average results of their high school. Within CA, HEIs can choose to give extra points to applicants with an underrepresented background to increase their participation in the program [8, 74].
Quota	In a quota system, seats are reserved for members of a particular target group, e.g. rural students, Indigenous students, or students from an underrepresented ethnic background. Quota can be used to improve access to HPE for underrepresented student groups, and to admit a student body which better represents the sociodemographic diversity in the country as a whole. Quota are used in e.g. Brazil [10, 75], India [76], Australia [44] and Japan [60].
Bonded Medical Places (BMP)	BMP is a scheme in which eligible applicants from certain regions (e.g. a rural region with a shortage of healthcare professionals), in return for guaranteed admission, sign a contract in which they agree to work in a rural/remote region for a number of years after completion of their studies. One of the goals of BMP is to reduce regional shortages of healthcare professionals and thereby improve access to healthcare. Research from Australia shows that the use of BMP also leads to an improved representativeness of the student population [44].
Lottery with extra tickets for target group members	The Netherlands used to have a (weighted) lottery-based admission system, based on high school GPA. In the past few years, lottery was not allowed, but it is likely to become legal again. We therefore included the option of extra tickets for target groups as a potential equitable admissions policy within a lottery procedure. The advantage of such a system would be that applicants who belong to underrepresented groups in HPE (and who are underrepresented in the applicant pool) have statistically higher odds of being admitted, thereby increasing the representativeness of the student population.

#### Stakeholders group

The stakeholders group is expected to consist of 30-50 people. We chose to invite persons and organizations who, in different capacities, are involved in (research of) either medical education, healthcare or higher education, or who represented potential target groups for equitable admissions. Stakeholders were recruited using purposive sampling [70], and invited via e-mail and telephone. The thirty-eight stakeholders who participated included (HPE) student and study associations, both at the university and national levels; national associations of healthcare professionals; representatives of possible equitable admissions target groups; researchers in the field of (higher) education in general or HPE in particular; high school deans and study counsellors; and representatives of the following organizations: hospital patient councils, a university hospital board; the Ministry of Education, Culture and Science, the Dutch Inspectorate of Education, the Social and Economic Council of the Netherlands, and the Advisory Committee on Medical Manpower Planning [*Capaciteitsorgaan*]. We also invited national patient organizations, but they declined to participate, e.g. due to time constraints.

All groups of participants included persons belonging to underrepresented groups in HPE.

### Procedure

**Fig. 1:**
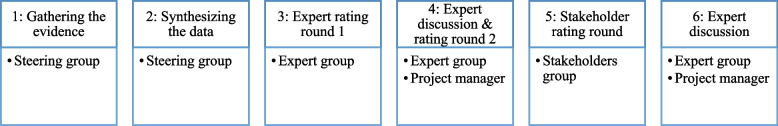
Research procedure

The FCM procedure consisted of the following steps (summarized in Fig. 1).

#### Step 1: gathering the evidence

The steering group acquired data to inform the proposed policies and target groups in four ways. Through retrospective cohort research we investigated which groups of students are underrepresented in university-based HPE programs as well as which groups of applicants have lower odds to be admitted. Next, an extensive review of equitable admissions policies and target groups used by universities all over the world was compiled by S.S. and L.M.. We conducted in-depth interviews with international experts (one from Scotland, one from Australia) to gain more insight into how these policies were developed in other countries. To gather more information on the history of, and most recent developments in, equitable admissions around the world, L.M. attended two international conferences (one on HPE – including on HPE selection research; one on access to higher education).

#### Step 2: synthesizing the data

The steering group summarized data in two documents. The main document explained the research procedure, the four proposed equitable admissions policies (Table 1) and ten potential target groups (Table 2). Table 1 contains the rationale for each of the four proposed policies (Contextualized Admissions, Quota, Bonded Medical Places, and lottery with extra tickets for target group members). Table 2 contains the summarized rationale for the inclusion of each target group. For each target group, the main document discussed what was known about the level of their underrepresentation and/or lower odds of admission (the main document – in Dutch – is available upon request). The appendix gave additional information on policies and target groups used around the world (see Supplementary Material 1 for a summary). According to the FCM, each individual policy and target group is defined as a ‘proposal’. The study documents emphasized explicitly that the research was not about the feasibility or practicability of proposals, but only about their suitability for the Dutch HPE context. This was done to prevent that people would rate proposals as unsuitable, simply because they are not legal and therefore currently not feasible. Additionally, the feasibility of policies is a concern which comes secondary to whether experts and stakeholders are open to them in the first place.

**Table 2:** Potential target groups for equitable admissions policies in the context of Health Professions Education in The Netherlands: definitions and rationale for inclusion

**Target group**	**Summary of rationale for inclusion (extensive rationale available upon request)**
Applicants from regions with a low participation rate in university education	Research by the authors (unpublished) shows that regions of the Netherlands with a low participation in university education bring forth very few HPE students.
Applicants from regions with a shortage of university-educated health professionals	Research by the authors (unpublished) shows that (mainly) rural regions of the Netherlands where there is a shortage of healthcare professionals, bring forth limited numbers and low proportions of HPE students compared to urban regions. Increased admission of applicants from these areas could lead in the long term to combatting healthcare shortages, as Dutch research shows that many medical specialists return to provide healthcare in their region of origin [77].
Applicants with a low or average socio-economic status (SES) background	SES is a key determinant of educational attainment and access to higher education [78]. Internationally, prior academic attainment has traditionally been the primary focus of selection into medical school [31]. Dutch HPE students disproportionately come from the highest SES groups. Almost 80% of HPE students who enrolled between 2016-2018 had at least one parent with a top-20% income. In comparison: this was only the case for 55% of all youth of the same age. More than 68% of HPE students belonged to the wealthiest 30% of households, which was the case for only 43% of their age-related peers. Logistic regression analysis showed that applicants with at least one parent in the top-10% wealth decile had significantly higher odds of admission (OR 1.28 [1.10–1.49]) than applicants whose parents belonged to wealth percentiles 0-70 [54].
Applicants with at least one parent on social welfare	Out of Dutch HPE students who enrolled between 2016-2018, 2.8% had at least one parent on social welfare, compared to 6.9% of their age-related peers [54].
Applicants without college/university-educated parents	It is estimated that approximately 23-24% of all university students in the Netherlands is a first-generation student. Amongst students with a so-called ‘non-western migration background’, this estimated percentage is 59% [79].
Applicants whose parents are not registered healthcare professionals	Applicants with at least one registered healthcare professional (HP) parent have significantly higher odds of admission in selection-based admission [54]. Students with HP parents are also overrepresented in HPE: In 2018, 25% of HPE students had at least one HP parent. This was the case for only 8.3% of their age-related peers [54].
Applicants with an underrepresented migration background	Students with a Turkish, Moroccan, Surinamese or Dutch Caribbean (TMSD) migration background make up only 5.7% of the Dutch HPE student population, despite making up 10.1% of their age cohort. In addition, TMSD applicants had significantly lower odds of admission (OR 0.72 [0.57–0.91]. This could not be explained by the socio-economic status of their parents [54].
Applicants who are asylum status holders (people with a recent refugee background who have received a (temporary) residence permit)	The odds of admission for asylum status holders are unknown. However, some general barriers to university admission for refugees are well-known: language proficiency [80], lack of information about the study, selection criteria and the admissions procedure [81], and their educational background which often differs from traditional applicants without a refugee history [82]. Refugees may be unable to bring their diplomas when they flee, making it harder to prove their qualifications to enroll in higher education [81, 83].
Men	The proportion of male HPE students decreased from 34.6% in 2011 to 28.6% in 2018 [54]. This is mainly due to lower application rates (between 2016-2018, men made up 31.6% of the applicant pool), but they were also disadvantaged in the selection procedure: women had significantly higher odds of being admitted (OR 1.35 [1.19–1.53].
Applicants with a visible or invisible disability	Approximately 17% of Dutch medical students have a visible or invisible disability (incl. dyslexia and dyscalculia) [84]. No significant differences were found in the odds of admission compared to applicants without a disability. However, international research points to several barriers for this target group. For example, they can encounter discrimination or stigmatization [85, 86], educational demands which make participation impossible [85], incorrect assumptions and stereotypes about disabilities [85], and lower expectations [86]. They may feel restricted to request certain accommodations, due to fear to be seen as less capable [86].

#### Step 3: expert rating round 1

In the first rating round, expert group members received the study documents via email and individually rated the suitability of each proposal in a form. Ratings ranged from 1 (‘totally unsuitable’) to 9 (‘totally suitable’). They were also asked for their opinion (free text) on equitable admissions policy in general, selection-based and lottery-based admission, and whether they wanted to propose additional policies or target groups. We analyzed the level of support for each proposal following the FCM guidelines (see Table 3), summarized the quantitative results and sent these to all experts one week before the expert discussion (Step 4).

**Table 3:** Score analysis, according to the formal consensus method guidelines

	**Median of ratings**		**Range of scores**
**Expert rating round 1**			
Proposal is appropriate	≥ 7	AND	[5–9]
Proposal is inappropriate	≤ 3.5	AND	[1–5]
Proposal is of uncertain appropriateness	[4-6.5] (undecided)	OR	All other situations (no consensus)
**Expert rating round 2**			
Proposal is appropriate (strong agreement)	≥ 7	AND	[7–9]
Proposal is appropriate (relative agreement)	≥ 7	AND	[5–9]
Proposal is inappropriate (strong agreement)	≤ 3	AND	[1–3]
Proposal is inappropriate (relative agreement)	≤ 3.5	AND	[1–5]
Proposal is of uncertain appropriateness (undecided)	[4-6.5]	AND	[1–9]
Proposal is of uncertain appropriateness (no consensus)	All other situations		

Ratings ranged from 1 (‘totally unsuitable’) to 9 (‘totally suitable’)

#### Step 4: expert discussion and rating round 2

A virtual meeting was held in which all 17 experts discussed their points of view. The meeting facilitator was not a part of the research team. Afterwards, experts again filled out the rating form. We analyzed the level of consensus.

#### Step 5: stakeholder rating round

As there were no proposals which were unanimously deemed appropriate (accepted) nor inappropriate (rejected) by the experts, the same documents were sent to the stakeholders via email. The stakeholders participated in one individual rating round, and returned their responses via email. Their quantitative responses were summarized using the mean, median and a bar chart summarizing all responses. Qualitative responses were anonymized and included in full. Both documents were sent to the expert group.

#### Step 6: expert discussion

Experts were invited to participate in one of two meetings to discuss the stakeholders’ feedback (due to scheduling issues, we needed to deviate from the FCM guidelines stating that all experts should attend the same meeting). One meeting was facilitated by a researcher who was not part of the research team, the other meeting by the project manager L.M.. Experts brought forward new suggestions for policies and target groups.

In the FCM, the main data used to determine the existence of consensus are the quantitative data: namely, the median and range of the ratings. The textual responses (free text) can provide additional information on why certain ratings were given, but come secondary to the ratings.

### Data analysis

IBM SPSS Statistics 28 and Excel 2016 were used to record textual responses and ratings, and to calculate medians and ranges of the ratings. The textual responses were organized by L.M. to represent distinct categories. These were reviewed by A.W., after which a few textual responses were re-categorized. Categorization was done as follows: opinions about equitable admissions policy in general were categorized as ‘positive’; ‘negative/not in favor/prefers only other policies’; or ‘mentions both advantages and disadvantages of equitable admissions’. Opinions on lottery and selection-based admission were categorized as ‘prefers lottery (weighted or unweighted)’; ‘prefers a combination of selection and lottery’; ‘prefers selection (under certain conditions); or ‘mentions advantages and disadvantages of both admission systems’. All results were discussed in team meetings and through written feedback on the initial manuscript.

The method, although evidence-based with regard to the proposals which were sent to participants, did allow participants to share their own views on these proposals. We therefore approached the study with a relativist paradigm [87], as we understood that there is no universal social reality which is valid for everyone. People have different perspectives, based on their social location and experiences, and this means they may interpret social facts differently. This results in multiple realities, rather than one absolute reality [87].

## Results

Supplementary Material 2 contains a summary of all quantitative results of the expert and stakeholder ratings. Table 4 summarizes which policies, target groups and combinations thereof had a median of at least 7 in at least one rating round.

**Table 4:** Proposals with a median of ≥ 7 in the expert and/or stakeholder groups

	**Median of expert ratings - Round 1**	**Median of expert ratings – Round 2**	**Median of stakeholder ratings**
**Policy**			
Contextualized Admissions (CA)	8	7	8
Lottery with extra tickets for target groups	8	7	6.5
**Target groups**			
Applicants with a low or average socio-economic status (SES) background	8	8	8
Applicants with at least one parent on social welfare	7	6	7
Applicants with an underrepresented migration background	7	8	8
Applicants who are asylum status holders	5	6.5	7
**Combinations of policies specific to different target groups**			
CA for applicants with a low or average socio-economic status (SES) background	7.5	7.5	8
CA for applicants with at least one parent on social welfare	8	5	8
CA for applicants without college/university-educated parents	5	5	7
CA for applicants with an underrepresented migration background	7	7.5	8
CA for applicants who are asylum status holders	5	7	7
Quota for applicants with an underrepresented migration background	2.5	1.5	7
Bonded Medical Places for applicants from regions with a low participation rate in university education	4.5	3	7
Bonded Medical Places for applicants from regions with a shortage of university-educated health professionals	7.5	5.5	8
Lottery with extra tickets for applicants from regions with a low participation rate in university education	7	5	4
Lottery with extra tickets for applicants with a low or average socio-economic status background	7	5	4
Lottery with extra tickets for applicants with an underrepresented migration background	7	7	7
Lottery with extra tickets for applicants who are asylum status holders	4	5	7
Bonded Medical Places for applicants from the Dutch Caribbean islands^a^		Combination was brought up in second expert meeting, 12 out of 17 experts (70%) were in favor	^a^

^a^The stakeholders group was not consulted on this option

The following section presents several written responses, which give an insight into the rationales provided for the ratings.

### Opinions about equitable admissions policy in general

A large majority of participants had a positive opinion about equitable admissions policy in general (13 out of 17 experts in the first round, 11 out of 17 in the second round, 31 out of 38 stakeholders). For example, the board of an association for health professionals and students (quoted with written informed consent) argued:

*“especially within health professions education, our experience is that there is little attention towards diversity and inclusion. We all experience this first hand and try to bring about a change. However, this is extraordinarily difficult and mentally draining, seeing our underrepresented position. Considering the fact that we are also ‘real foreigners’ (coming from the [Dutch Caribbean] islands, moved to The Netherlands to study at age 18), we are an even smaller group with even less networks and support. We would appreciate every policy or extra support to make our opportunities approach those of our peers.”*

Three experts (second round) and two stakeholders wrote about advantages as well as disadvantages of equitable admissions policies, and only a minority (one expert, four stakeholders) was not in favor of any policy. Those who were not in favor emphasized that they would rather see other efforts to increase the diversity of the student population, e.g. improved recruitment and outreach efforts, and inclusive promotion materials. They were therefore in support of the end goal, but not of this particular method to achieve it. For example, one expert argued:

*“I continue to hold the opinion that inequality of opportunity needs to be tackled at the preliminary stage, meaning during the moment of school and career choice, by proactive and stimulating outreach, buddy programs, etcetera and NOT by reparation-attempts during selection. The selection procedure itself should be designed as objective and fair as possible so that everyone has the same opportunities (and there remains a lot to be gained there). Only for asylum status holders I would like to make an exception, since they are often unreachable in the preliminary stage.”*

### Opinions about Contextualized Admissions

The majority of experts (median first round: 8, second round: 7) and stakeholders (median: 8) supported the use of Contextualized Admissions (CA). One expert wrote:

*“Very suitable because it takes into account the fact that not everyone has the same starting position and not everyone has the same social and cultural capital.”*

A stakeholder argued:

*“We need to look at the bigger picture and not settle on the real grade that someone achieves, but look at someone’s potential instead.”*

Participants who were in favor of CA saw it as an advantage that *all* applicants would go through the CA process, rather than only those applicants belonging to target groups. They argued this may help to avoid ‘labelling’ or ‘stigmatizing’ target populations. There was majority support (all medians: 7 or 8) for using CA to increase the matriculation of applicants from a low or average SES background, with parents who receive social welfare, with an underrepresented migration background, and asylum status holders. However, the range of scores was 1-9, meaning there was no official consensus according to the FCM guidelines. This is because for official consensus, the range of ratings should fall between 5-9 (see Table 3).

### Opinions about quota and lottery with extra tickets for target groups

Quota (median experts: 5, stakeholders: 6) and lottery with extra tickets for target groups did not receive majority support from either group. The potential issue of ‘stigma’ for target populations was one of the main objections against quota. Lottery with extra tickets for target groups was supported by experts in the first (median: 8) and second round (median: 7), but the stakeholder round showed no majority support for this option (median: 6.5). Most low ratings were not accompanied by written feedback, therefore it is uncertain why participants did not support this option. The few comments which were provided alongside low ratings show either a preference for random lottery, without extra tickets for any group, or a preference for selection rather than any form of lottery. Nevertheless, most of the written feedback which was given for this option was positive. The only target group for which both groups showed majority support in combination with lottery with extra tickets, was applicants with an underrepresented migration background (median of 7 in all rating rounds).

### Opinions about Bonded Medical Places

For Bonded Medical Places (BMP) (first expert round: 5, second round: 4, stakeholders: 4.5), objections were that it would limit the individual freedom of students, and that most applicants are too young to sign a BMP contract, as their ambitions may change. However, during the final expert meeting, several expert group members proposed to use BMP for Dutch Caribbean applicants, in light of the shortage of healthcare professionals on the Dutch Caribbean islands. Moreover, they discussed that Dutch Caribbean applicants were disadvantaged in the current selection procedure. As this combination was introduced in the final expert meetings, stakeholders were not consulted on this option. 12 out of 17 experts were in favor of this combination.

### Responses regarding the different target groups

Support for the ten proposed target groups differed greatly. The highest ratings (median: 8 in all rounds) were given to applicants with a low or average socio-economic status background, and to applicants with an underrepresented migration background (experts: 7-8, stakeholders: 8). Participants argued that low- and average SES applicants currently face disadvantages, not just in education and selection but also in other areas of life, and that having equitable admissions for this target group would not only be fair towards them, but would also be good for patients with a low SES (e.g. as they “disproportionately need healthcare” and “deserve to have doctors who can connect with their living situation”). Furthermore, they argued that it is possible to delineate this group properly with low odds of incorrectly including or excluding people. Participants argued that applicants with an underrepresented migration background could contribute to better healthcare for patients, based on their language skills, cultural knowledge, and the feeling of recognition by the patient. It was also suggested that due to their possible experience with discrimination, prejudice or stereotypes, they would be able to empathize more with patients who experience the same issues. Furthermore, participants argued that increased representation and inclusion within the medical workforce was a goal in itself that should be achieved.

Some participants gave low ratings to target groups on the basis that equitable admissions for these applicants could ‘feel unfair’ towards applicants who did not belong to target groups. Others wrote that people could ‘feel stigmatized’ by being made a target group for equitable admissions – suggesting that some participants gave low ratings which were ‘well-meant’ (to avoid stigma), rather than indicating opposition to improved representation of the target group in HPE. The lowest ratings (medians: 3.5-4) were given to applicants whose parents are not registered healthcare professionals. Participants argued for example that it is normal for children to follow in the footsteps of their parents’ careers, that HPE programs should no longer use selection instruments through which those with a medical network have an (unintended) advantage, but that equitable admissions focused on this target group would be unfair towards the children of healthcare professionals.

Several additional target groups were suggested by experts and stakeholders. The most often proposed group was members of the LGBTQIA+ community, or a particular group within this community, e.g. transgender people. However, there was no majority support for any additional group. A few participants recommended to take intersectionality [88] into account, as persons belonging to multiple target groups may face cumulative disadvantages in the educational system.

### Opinions about lottery and selection

On the topics of lottery and selection, there was no consensus. The rationales provided in favor of a lottery focused on perceived equality (“everybody has the same chance”), reduced stress for applicants, easiness and affordability, and an assumed increase in diversity of the student population. Arguments in favor of selection (under certain conditions, e.g. non-biased selection instruments) were e.g. an assumed improved quality of healthcare, the ability to select applicants on the basis of more than only cognitive instruments, and the ability to admit applicants with the highest odds of graduating. There were as many stakeholders (n=10) who preferred lottery as there were stakeholders who preferred selection, and no stakeholders indicated a preference for a combination of the two. In the expert group, twelve members supported such a combination. They were particularly in favor of a method in which first one or more selection instruments would be used to evaluate applicants. The highest scoring group would then be admitted, the lowest scoring group rejected, and in the middle group, a lottery would take place. The assumption was that lottery in the middle scoring group (where participants have similar aptitude) would lead to more diversity.

### Other responses

Some experts voiced concerns about practical difficulties of designing or implementing equitable admissions, and their judicial illegality. These concerns sometimes took dominance during expert meetings, even though the research was not about feasibility, but suitability. Furthermore, some participants had entirely different ideas of the (origins of the) problem, and about the required solutions. For example, some argued that as long as the chosen selection instruments were valid and unbiased, they considered the outcome of the selection fair. Others argued that the main origins of educational inequality are based in early life, and should not be ‘fixed’ at the moment of university admission.

Numerous suggestions were given for additional policies to increase the representativeness of HPE students. Amongst those, the top three suggestions pertained to improved guidance of underrepresented groups in HPE at an early stage (in primary/secondary education), to increase their odds of becoming eligible to apply; inclusive recruitment and outreach efforts to increase diversity in the applicant pool; and the use of unbiased selection instruments and procedures.

## Discussion

Using the FCM, this study investigated the level of societal and institutional support in The Netherlands for different equitable admissions policies and target groups in Health Professions Education programs.

We found majority support for several proposals. However, the range of scores was 1-9, meaning there was no consensus. First, we found majority support in the expert and stakeholder group for the target groups ‘applicants with a low or average socio-economic status’ and ‘applicants with an underrepresented migration background’. The majority was also in favor of Contextualized Admissions, especially when used to increase enrolment of applicants with a low or average socio-economic status, with an underrepresented migration background, and asylum status holders. Both groups supported lottery with extra tickets for applicants with an underrepresented migration background. The expert group also proposed to use Bonded Medical Places for applicants from the Dutch Caribbean islands. Although the high ratings for certain proposals indicate broad support for equitable admissions, many proposals did not receive support, despite the evidence demonstrating their relevance. This shows a gap between available evidence, and institutional and societal support.

Furthermore, some proposals received high ratings in one group, but low ratings in the other group (e.g. quota for applicants with an underrepresented migration background – which showed a median rating of 1.5 in the second expert round, but a median of 7 amongst stakeholders. This suggests that although all participants received the same study documents, the way in which they interpreted or evaluated the evidence therein, differed significantly. As we approached the study with a relativist paradigm [87], understanding that people have different perspectives based on their social location and experiences, we expected that they could interpret social facts differently.

The findings in this study are of practical relevance to those in the Dutch higher education sector who wish to improve equity in admissions, and to readers in other countries who wish to investigate the societal and institutional support for equitable admissions in their own context, using an evidence-based method. Our application of the FCM in the area of new higher education admissions policies may be useful for researchers in other countries where equity principles are not (widely) used in admissions decisions. Examples include China and South Korea, where educational inequities are large, and holistic review is not standard [71, 89–93] but only used by some top universities for a portion of their applicants [94].

The remainder of the discussion will focus on our recommendations for the Dutch HPE context, and offer reflections on the FCM in the context of equitable admissions.

### Recommendations for the Dutch HPE context

Currently, Dutch selection procedures assess applicants’ achievements (e.g. test scores, GPA, extracurricular activities) without being able to weigh them with regard to the context in which they were achieved. This is a limitation, as many assessment instruments are “likely to be better indicators of achievement and potential if their implementation acknowledges contextual factors (such as educational context and personal circumstances) (…) and considering other additional information should also help to ensure that all applicants have equal opportunity to demonstrate relevant achievements and potential” [95]. Since this study found majority support for Contextualized Admissions and lottery with extra tickets for a number of target groups, legalization of these policies could be the first step towards achieving a representative student population and improving equity in admissions. More research is necessary to investigate what the best evidence-based methods are for implementing CA in practice. Expertise from abroad could aid in effective, evidence-based design and professional implementation of CA [8, 96]. As The Netherlands has a centralized applications service (called ‘Studielink’), and a national bureau of statistics with an immense amount of data on each citizen, there is much that can be learned from international expertise – such as the recommendation to use administratively verified individual-level metrics (like low household income) rather than area/neighborhood-level metrics [97].

Legalization of Bonded Medical Places for applicants from the Dutch Caribbean islands could improve the representativeness of the student population and help to combat regional inequities in access to healthcare and HPE between the European and Caribbean parts of the Kingdom of The Netherlands. Experts in the area of BMP in Australia could be consulted in the development of evidence-based methods to implement BMP effectively in The Netherlands. Considering the shortages of health professionals in mainly rural parts of The Netherlands, BMP for applicants from these regions could contribute to the goal of equitable access to healthcare as well.

We suggest that, based on the available evidence, it could be relevant to increase advocacy for and awareness about the need for equitable admissions, which could increase the support for the legalization of additional policies and target groups. For example, in Scotland, SCAPP (Scotland’s Community of Access and Participation Practitioners) members from universities, colleges, skills, voluntary and public sector organisations form a practitioners’ network “to support the development and professionalisation of a strong widening access and participation community” [96]. Furthermore, as several experts and stakeholders suggested, it could be appropriate to take intersectionality [88] into account when designing equitable admissions procedures, as persons who belong to multiple underrepresented groups may have faced cumulative disadvantages on the route to higher education. An intersectional approach could include *structural competency,* a healthcare paradigm which aims to address “the hierarchies that produce unjust health conditions” [49], rather than placing too much emphasis on the individual. Applying this paradigm to admissions means that “understanding the structures that place one at a disadvantage expresses a knowledge base or competency in how structures produce and reproduce power; this competency should be regarded as a unique, valuable, hard-earned merit” [33]. The lived experiences of HPE applicants belonging to disadvantaged groups can improve the connection with patients and colleagues, an essential element of achieving equitable health outcomes [33]. Furthermore, their perspectives and voices are essential in the development of policies in HPE that aim to deal with questions of equity, anti-racism and diversity [50, 51].

As a number of experts and stakeholders argued as well, equitable admissions procedures such as CA can only provide a partial answer to unequal access to higher education, next to e.g. outreach, support at university, and into the labour market or continued studies [8].

### Reflections on the lottery versus selection debate

Some participants argued that the use of (partial) lottery would lead to fairness and increased diversity. These ideas also exist elsewhere, in favor of a random lottery for everyone who meets a minimum test score or GPA [34], or a hybrid system where a lottery is held after a selection procedure [35]. However, there is no evidence that this would lead to a representative population,. In the Dutch context, a hybrid weighted lottery-and-selection procedure also placed underrepresented groups in HPE at a disadvantage in terms of their odds of admission. Although clear differences are seen with regard to diversity between lottery and selection-based admission, a random lottery would not result in a student population which is as diverse as their age peers [54, 56]. Furthermore, no (sub)group of applicants favors (weighted or random) lottery [65].

In the U.S. context, Baker and Bastedo [98] proved through large-scale simulations that most lotteries would result in *lower* proportions of students from low-income backgrounds and students of color. They argue that “lotteries based on some combination of GPA and test scores do not automatically create a more equitable class [because] the measures themselves are inequitable” (p. 143). A (partial) use of lottery, without an in-built equity instrument, is thus unlikely to result in a representative student population. Therefore, we do not recommend the use of (partial) lottery-based admission as long as the applicant pool is not representative of the patient population they may serve as medical professionals in the future. However, if lottery is used, we recommend HEIs to ensure that members of underrepresented groups in HPE matriculate at higher rates. This can be done by using (stratified) quota, and/or by awarding them with additional lottery tickets. Although this type of lottery did not receive majority support in our study (due to the stakeholders’ median of 6.5), the scientific evidence warrants advocacy for the use of admission instruments which can achieve a representative student population. Furthermore, research by the CPB Netherlands Bureau for Economic Policy Analysis shows that it is possible to use selection algorithms to select a representative population *and* to maintain effectivity, thereby increasing representation amongst Medicine students and resulting in better outcomes than a random lottery in terms of study completion [99].

### Reflections on the Formal Consensus method

The FCM enabled us to investigate the current levels of support for several equitable admissions policies and target groups. This section highlights our critical reflection on the use of FCM, to enable researchers, policy makers and others to evaluate whether the FCM could be an appropriate method for investigating the support levels for equitable admissions policies in their context.

First, numerous low ratings were based on *practical concerns*, rather than a disagreement with (underlying principles or effects of) particular proposals. As many experts have years of experience with (the development and implementation of) current admissions, we could have anticipated that their frame of reference is influenced by practice-based considerations. Other researchers could take this into account. This is important, because the low ratings of a few individuals resulted in a range of ratings between 1-9, meaning that no proposal was deemed having a consensus according to the FCM guidelines.

Furthermore, although participants were presented with scientific *evidence*, they were thereafter asked for their *opinion*. In some cases, participants wrote that the evidence changed their perspectives on selection, lottery, and other topics. However, other participants gave more weight to other values underlying opinions on the research subjects. It is possible, however, that since equitable admissions procedures have never been legal in The Netherlands, it becomes more difficult to appreciate unknown potential solutions to inequality of opportunity.

Finally, when the FCM is used for medical protocols (its original purpose), the ‘problem’ may be clear to all. However, in our research, there was no shared view of the *problem definition*. Without a consensus on the problem (or whether there *is* a problem at all), it is hard to reach a consensus on potential solutions. Therefore, readers in other contexts may need to ensure at the beginning of the project that there is a commonly shared and agreed upon problem definition. We recommend to make this a formal part of the standard FCM procedure.

### Strengths and limitations

A strength of the research is that we worked with a highly diverse range of organizations, institutions and groups, including from persons outside of the health sector, as recommended by Abubakar et al. [100] in The Lancet Series on Racism, Xenophobia, Discrimination and Health, to address the systems that have thus far contributed to the underrepresentation of certain demographic groups in Dutch HPE. However, a limitation is that not all invited experts and stakeholders decided to participate, leaving especially patient representatives underrepresented in the research. Since diversity amongst healthcare providers is associated with improved healthcare to underserved patients, it is possible that stakeholder ratings would have been different on average if more patient organizations had participated. Next to time constraints, limited awareness about the impact of selection practices on the healthcare workforce and thus healthcare practice may have kept patient representatives from participating. We expect that this awareness may be heightened based on new research [56, 63].

Several target groups which received low ratings, were (potentially) underrepresented amongst the study participants. For example, men were underrepresented in both groups. This may be a partial explanation for the low ratings given for men as a target group, despite the evidence presented that they are underrepresented in HPE (<30% of all students) and face bias in admissions [54]. With regard to applicants from regions with a shortage of university-educated health professionals, a limitation is that invited stakeholders with known expertise in this area chose not to participate in the study. We do not know to what extent our participants live in, or come from, such regions, nor whether potential underrepresentation in this study could be an explanation for the lack of majority support for this target group.

Since the proposed equitable admissions procedures have never been legal in The Netherlands, there are no content experts with practical experience on this matter in the Dutch HPE context. We therefore had to rely mainly on international literature and experts on equitable admissions policies. At the time of the research, we could not find literature in which lottery with extra tickets for underrepresented groups or other forms of ‘equitable’ lotteries were studied empirically or theoretically. It may be possible that the limited evidence base resulted in the lack of majority support amongst stakeholders for this proposed type of lottery.

Finally, based on the above limitations, and the unique admissions context and history of The Netherlands, we cannot generalize empirical findings to other contexts.

## Conclusion

The support base for the abovementioned policies and target groups provides a foundation for working towards legislative changes in The Netherlands, to make these forms of equitable admissions policy legally possible. There remains a gap between the available evidence for the need for other policies and target groups, and the institutional and societal support for them. Additional research, advocacy and awareness may be necessary to promote support for more types of policy and target groups which could benefit equity in admissions, lead to a more representative student population, and benefit the future provision of excellent, equitable healthcare to all patient populations.

## Supplementary Information

Supplementary Material 1.

## Data Availability

The data that support the findings of this study are not publicly available due to them containing information that could compromise research participant privacy and consent. Anonymized data, non-traceable to participants, are available from the corresponding author on reasonable request.
