# Supplementary material for: Investigating the support for equitable admissions policies in health professions education: the Formal Consensus method
Source: BMC Med Educ. 2024 Oct 17;24:1157. doi: 10.1186/s12909-024-06049-y (PMC11484433; doi:10.1186/s12909-024-06049-y)
Supplement: Supplementary file 1 — Supplementary Material 1. [file 12909_2024_6049_MOESM1_ESM.docx]

**Supplementary Material 1: Examples of the use of Contextualized Admissions, Bonded Medical Places and quota around the world**

*Abbreviations:***IMD**: Indices of Multiple Deprivation. These classify the relative deprivation of small areas. The lower the IMD, the more deprived the area is.

**POLAR**: Participation of Local Areas. This indicates the rate at which young people join higher education.

**UCAT Bursary**: Financial help for individuals who cannot pay for the University Clinical Aptitude Test (UCAT). The UCAT is used as an admissions test for medicine courses in the UK.
 **Examples of universities using contextual admissions**

| **University policy** | **Target groups** |
| --- | --- |
| **University of California, Los Angeles (UCLA), United States** UCLA’s comprehensive admission review process is based upon 13 criteria, which include both quantitative and qualitative, academic and personal accomplishments by the student along with consideration of the context of those accomplishments and opportunities afforded to the student. See: <https://admission.ucla.edu/apply/freshman/freshman-requirements/application-review-process> | All applicants are reviewed in a comprehensive manner. The UCLA website states: “We consider all achievements, both academic and non-academic, in the context of the applicant’s actual opportunities. Readers base their assessments on how fully the applicant has taken advantage of those opportunities. To gauge the strength of the high school curriculum, we look at the school’s resources; the availability of honors, AP, and IBHL courses; and the total number of college preparatory courses available. When they can, readers compare the achievements of students in the same applicant pool who attended the same high school. These applicants are likely to have similar opportunities to achieve.” |
| **Cardiff University, United Kingdom:**  Weighted score (using yes/no metrics) is given for each of the WA indicators (on the right) to create an overall contextual score. Students with scores of 100 or above qualify as a Widening Access target group student. These scores give students extra credits, and are used to determine who will be invited for interviews. | Low-income household; Living in low POLAR area; Living in low IMD area; Free school meals; In care; First-generation higher education student. |
| **Hulk York University, United Kingdom:**  WA target groups will be offered an interview at a lower academic performance (AAB) than generally required. Students with a lower academic performance (ABB) can join a pathway year. | Living in low POLAR area; First-generation student; Eligible for UCAT Bursary. |
| **Flinders University, Australia:**  Indigenous applicants will get an interview without having to sit a test. | Individuals who identify and are accepted as Aboriginal or Torres Strait Islander. |

**Examples of universities using Bonded Medical Places (BMP)**

| **University policy** | **Target groups** |
| --- | --- |
| **UNSW Sydney, Australia:**  At least 29% of the seats for domestic students are for rural applicants. These students are required to serve for 3 years in a rural area, within an 18-year period. | Applicants living in a rural area for at least five consecutive or ten cumulative years since the age of 5. |
| **Australian National University, Australia:**  28% of seats are reserved for rural applicants. These students are required to serve for 3 years in a rural area, within an 18-year period. | Applicants living in a rural area for at least five consecutive or ten cumulative years. |

**Examples of universities using quota**

| **University policy** | **Target groups** |
| --- | --- |
| **Aston University, United Kingdom:**  40% of the places are reserved for Widening Access target groups. | Living in low POLAR 4 area; First-generation higher education student; Eligible for UCAT bursary; Free school meals; In care; Underrepresented group: Gypsy, Roma, Traveller communities, refugees, children of military families. |
| **Flinders University, Australia:**  28% of places are reserved for rural applicants  5 places are reserved for Aboriginal and/or Torres Strait Islander applicants. | Applicants living for 5 years in a rural area (or cumulatively 10 years); Aboriginal and/or Torres Strait Islander applicants. |
| **Queens University, Canada:**  10 places are reserved for students who identify as Black or Indigenous. These students do not have to sit the Medical College Admission Test. | Students who identify as Black or Indigenous. |
| **University of Ottawa, Canada:**  7 seats are reserved for students who identify as Indigenous.  2 seats are reserved for individuals with low socioeconomic status. | Students who identify as Indigenous; From low-income household. |
| **Most universities in Japan:**  The majority of Japanese universities works with quotas for rural students. In some universities, students are required to serve in rural areas after their graduation. | Rural applicants |

**Supplementary Material 2: Policies and target groups with expert and stakeholder ratings**

|  | **Expert ratings, round 1** | | **Expert ratings round 2** | | **Stakeholder ratings** | |
| --- | --- | --- | --- | --- | --- | --- |
|  | **Median** | **Range^c^** | **Median** | **Range** | **Median** | **Range** |
| **How suitable do you find the following equitable admissions policies for the Dutch context?** | | | | | | |
| Contextualized Admissions | 8 | 1-9 | 7 | 1-9 | 8 | 1-9 |
| Quota | 5 | 1-9 | 5 | 1-9 | 6 | 1-9 |
| Bonded Medical Places | 5 | 1-9 | 4 | 1-9 | 4.5 | 1-9 |
| Lottery with extra tickets for target groups | 8 | 1-9 | 7 | 1-9 | 6.5 | 1-9 |
|  | | | | | | |
| **How suitable do you find the following target groups for equitable admissions policy in the Dutch context?** | | | | | | |
| Applicants from regions with a low participation rate in university education | 5 | 1-9 | 4 | 1-9 | 5 | 1-9 |
| Applicants from regions with a shortage of university-educated health professionals | 7 | 1-9 | 5 | 1-9 | 5 | 1-9 |
| Applicants with a low or average socio-economic status background | 8 | 1-9 | 8 | 1-9 | 8 | 1-9 |
| Applicants with at least one parent on social welfare | 7 | 1-9 | 6 | 1-9 | 7 | 1-9 |
| Applicants without college/university-educated parents | 5 | 1-9 | 5 | 1-9 | 6 | 1-9 |
| Applicants whose parents are not registered healthcare professionals | 4 | 1-9 | 3.5 | 1-9 | 4 | 1-9 |
| Applicants with an underrepresented migration background | 7 | 1-9 | 8 | 1-9 | 8 | 1-9 |
| Applicants who are asylum status holders | 5 | 1-9 | 6.5 | 1-9 | 7 | 1-9 |
| Men | 4 | 1-9 | 4.5 | 1-9 | 5 | 1-9 |
| Applicants with an (in)visible disability | 3.5 | 1-9 | 4 | 1-9 | 6 | 1-9 |
| **How suitable do you find each different type of policy for each of the different target groups in the Dutch context?** | | | | | | |
| ***Contextualized Admissions*** |  |  |  |  |  |  |
| Applicants from regions with a low participation rate in university education | 7 | 1-9 | 4 | 1-9 | 3 | 1-9 |
| Applicants from regions with a shortage of university-educated health professionals | 5 | 1-9 | 3.5 | 1-9 | 3 | 1-9 |
| Applicants with a low or average socio-economic status background | 7.5 | 1-9 | 7.5 | 1-9 | 8 | 1-9 |
| Applicants with at least one parent on social welfare | 8 | 1-9 | 5 | 1-9 | 8 | 1-9 |
| Applicants without college/university-educated parents | 5 | 1-9 | 5 | 1-9 | 7 | 1-9 |
| Applicants whose parents are not registered healthcare professionals | 4 | 1-9 | 2 | 1-9 | 3 | 1-9 |
| Applicants with an underrepresented migration background | 7 | 1-9 | 7.5 | 1-9 | 8 | 1-9 |
| Applicants who are asylum status holders | 5 | 1-9 | 7 | 1-9 | 7 | 1-9 |
| Men | 4.5 | 1-9 | 3.5 | 1-9 | 2 | 1-9 |
| Applicants with an (in)visible disability | 3 | 1-9 | 3.5 | 1-9 | 6 | 1-9 |
|  |  |  |  |  |  |  |
| ***Quota*** |  |  |  |  |  |  |
| Applicants from regions with a low participation rate in university education | 3 | 1-9 | 1.5 | 1-9 | 2.5 | 1-9 |
| Applicants from regions with a shortage of university-educated health professionals | 2.5 | 1-9 | 1.5 | 1-9 | 3 | 1-9 |
| Applicants with a low or average socio-economic status background | 3 | 1-9 | 1.5 | 1-8 | 4 | 1-9 |
| Applicants with at least one parent on social welfare | 2 | 1-9 | 1 | 1-9 | 3 | 1-9 |
| Applicants without college/university-educated parents | 1 | 1-9 | 1 | 1-8 | 2.5 | 1-9 |
| Applicants whose parents are not registered healthcare professionals | 1 | 1-9 | 1 | 1-5 | 2 | 1-9 |
| Applicants with an underrepresented migration background | 2.5 | 1-9 | 1.5 | 1-8 | 7 | 1-9 |
| Applicants who are asylum status holders | 3 | 1-9 | 3 | 1-9 | 5 | 1-9 |
| Men | 2.5 | 1-9 | 2 | 1-9 | 3 | 1-8 |
| Applicants with an (in)visible disability | 1 | 1-9 | 1 | 1-9 | 4 | 1-9 |
|  |  |  |  |  |  |  |
| ***Bonded Medical Places*** |  |  |  |  |  |  |
| Applicants from regions with a low participation rate in university education | 4.5 | 1-9 | 3 | 1-9 | 7 | 1-9 |
| Applicants from regions with a shortage of university-educated health professionals | 7.5 | 1-9 | 5.5 | 1-9 | 8 | 1-9 |
| Applicants with a low or average socio-economic status background | 3 | 1-9 | 2.5 | 1-9 | 1 | 1-8 |
| Applicants with at least one parent on social welfare | 2.5 | 1-9 | 1 | 1-9 | 1 | 1-8 |
| Applicants without college/university-educated parents | 1.5 | 1-9 | 1 | 1-9 | 1 | 1-6 |
| Applicants whose parents are not registered healthcare professionals | 1.5 | 1-9 | 1 | 1-9 | 1 | 1-8 |
| Applicants with an underrepresented migration background | 2.5 | 1-9 | 3 | 1-9 | 2 | 1-9 |
| Applicants who are asylum status holders | 1.5 | 1-9 | 2 | 1-9 | 2 | 1-8 |
| Men | 2 | 1-9 | 1 | 1-9 | 1 | 1-8 |
| Applicants with an (in)visible disability | 1.5 | 1-9 | 1 | 1-9 | 1 | 1-9 |
|  |  |  |  |  |  |  |
| ***Lottery with extra tickets for target groups*** |  |  |  |  |  |  |
| Applicants from regions with a low participation rate in university education | 7 | 1-9 | 5 | 1-9 | 4 | 1-9 |
| Applicants from regions with a shortage of university-educated health professionals | 6.5 | 1-9 | 4.5 | 1-9 | 2 | 1-9 |
| Applicants with a low or average socio-economic status background | 7 | 1-9 | 6 | 1-9 | 5 | 1-9 |
| Applicants with at least one parent on social welfare | 6.5 | 1-9 | 4 | 1-9 | 5 | 1-9 |
| Applicants without college/university-educated parents | 5 | 1-9 | 3 | 1-9 | 5 | 1-9 |
| Applicants whose parents are not registered healthcare professionals | 3 | 1-9 | 1 | 1-8 | 3 | 1-9 |
| Applicants with an underrepresented migration background | 7 | 1-9 | 7 | 1-9 | 7 | 1-9 |
| Applicants who are asylum status holders | 4 | 1-9 | 5 | 1-9 | 7 | 1-9 |
| Men | 2 | 1-9 | 1 | 1-9 | 2 | 1-9 |
| Applicants with an (in)visible disability | 1 | 1-9 | 1 | 1-9 | 4 | 1-9 |

Open questions in the rating form were:

1. What is your position, in general, regarding equitable admissions policy policy?

2. What is your position, in general, regarding selection and lottery?

3. Are there any other target groups that you think should be eligible for equitable admissions policy in The Netherlands and/or at your university?

4. Is there any other type of policy that is not included in this document, that could potentially be suitable to increase the representativeness of HPE student populations? If yes, please explain what this policy would entail and at which target group(s) this policy should be aimed.
